# An insight into the suspected HbA2' cases detected by high performance liquid chromatography in Pakistan

**DOI:** 10.1186/1756-0500-4-103

**Published:** 2011-04-05

**Authors:** Maliha Nusrat, Bushra Moiz, Amna Nasir, Mashhooda Rasool Hashmi

**Affiliations:** 1 Department of Pathology and Microbiology, The Aga Khan University Hospital, Stadium Road, Karachi, Pakistan

## Abstract

**Background:**

Hemoglobin A2' (delta 16 Gly → Arg) is globally the commonest delta chain variant of HbA2. It is clinically and hematologically silent but its sole importance lies in the underestimation of HbA2 quantity during the workup of β-thalassaemia trait. High performance liquid chromatography (HPLC) identifies it as a small S-window peak with a mean retention time of 4.59 ± 0.03 minutes. This study aims at describing the frequency of detection of HbA2' by HPLC in Pakistan and its confirmation at a molecular level. Potential HbA2' cases were identified by a retrospective review of 10186 HPLC chromatograms in year 2006. Prospective samples were collected for polymerase chain reaction (PCR) amplification, restriction digestion and nucleotide sequencing.

**Findings:**

One hundred and ninety two potential cases (1.89%) of HbA2' were detected on HPLC, having mean retention time of 4.59 ± 0.05 minutes. Sixty four (0.6%) new cases were suspected of having co-existing β-thalassaemia trait when the quantity of S-window peaks was taken into account. Thirteen samples with presumed HbA2' on HPLC were subjected to molecular analysis and the said mutation (δ 16 GGC → CGC) was not detected in any sample.

**Conclusions:**

It is concluded that diagnosis of HbA2' on HPLC alone is not justified, as evidence of the presence of this delta chain variant in Pakistani population is yet to be proven. Such small S-window peaks should be either disregarded or confirmed at molecular level, and only then should influence the diagnosis of β-thalassaemia trait. Further studies are required to determine the true nature of these peaks.

## Background

Hemoglobin (Hb) A2', also called HbB2 or HbA2δ', is globally the commonest δ chain variant of HbA2. It was first discovered in 1958 in black community of Gulla James Island [1]. Since then it has been reported with increasing frequency among blacks with prevalence being 2.16% in Mauritania (Northwestern Africa) [2], 2.7% in Dogon (Mali) [3] and 18% in Herero (South Africa) [4]. Recently, this hemoglobinopathy has been observed in two Brazilian Caucasian women [5], and in white British [6].

HbA2' is a clinically silent hemoglobinopathy that results from modification of δ globin gene (GGC → CGC) substituting glycine for arginine at codon 16 [3]. This genetic disorder has been detected in homozygous [7] and heterozygous states [8], and may also be co-inherited with HbS [9], β-thalassaemia traits [10] and other minor hemoglobinopathies. The clinical significance of identification of HbA2' is in the detection of co-existing β-thalassaemia trait as, in the presence of HbA2', such individual might present with normal HbA2 levels resulting in under diagnosis of thalassaemia minor [11]. Hence, it is important to screen patients for HbA2'. Alkaline electrophoresis has limited resolution in this regard [12] and though detection by iso-electric focusing is easy, it lacks precise quantification [12]. On the other hand, microcolumn chromatography can accurately measure total HbA2 level without identifying HbA2'[6]. In contrast to these diagnostic tools, high performance liquid chromatography (HPLC) not only senses HbA2' as it elutes in the 'S' window (retention time of 4.59 ± 0.03 minutes) but also quantifies it which is usually in proportion of 1.2% of total hemoglobin [13].

Our clinical laboratory started using HPLC for detection of hemoglobinopathies in year 2000 and though minor peaks in S window were observed frequently, they were largely ignored as unexplained peaks. Recent literature review raised the possibility of them representing HbA2' [6,14]. Although HbA2' has never been reported in Pakistan, the possibility of a rare silent mutation existing in the country without being reported was very likely owing to the fact that limited research occurs here.

The objectives of the study were to evaluate the frequency of detection of small S-window peaks representative of HbA2' cases by HPLC and to confirm the presence of HbA2' mutation at a molecular level in Pakistani population.

### Hypothesis

Small S-window peaks on chromatography are secondary to the presence of HbA2' in the blood samples.

## Methods

Variant β-thalassaemia short program (Bio-Rad Laboratories, Hercules, CA, USA) utilizing the principle of cation exchange high performance liquid chromatography was used for detection of hemoglobin variants. We retrospectively reviewed all HPLC chromatograms for the year 2006. To define a diagnostic criteria for HbA2', we identified S-window peaks of <4% as potential cases [14]. Peaks of 1-2% were considered to have HbA2' trait [14] while a diagnosis of homozygous A2' was made when HbA2' was >2% with absent HbA2 [13]. We also included samples with peaks less than 1% in the analysis.(Table 1) Co-inheritance of HbA2' and β-thalassaemia (double heterozygote) was suggested where sum of HbA2 and HbA2' was greater than 3.5% of total hemoglobin [13].

**Table 1:** Analysis of 192 samples with suspected HbA2' with varying S-window peaks.

S-Window Peaks	Total Cases n (%)	Retention Time Range (min) Mean ± SD	HbA2* Range (%) Mean ± SD	Cases with HbA2 > 3.5% n (%)	Cases with HbA2+suspected HbA2'>3.5% n (%)	Additionally suspected β-thalassaemia minor n (%)
**<1%**	123 (64.2)	4.33-4.62 4.59 ± 0.04	1.0-7.0 3.22 ± 1.41	27 (22.6)	47 (39.5)	20 (16.9)
**1-2%**	63 (32.6)	4.32-4.62 4.59 ± 0.05	1.3-6.3 3.07 ± 1.08	10 (15.9)	51 (81)	41 (65.0)
**>2%**	6 (3.1)	4.44-4.61 4.56 ± 0.06	2.3-5.2 3.67 ± 1.05	3 (50)	6 (100)	3 (50)

**All cases**	**192(100)**	**4.32-4.62** **4.58 ± 0.05**	**1.0-7.0** **3.19 ± 1.30**	**40**	**104**	**64**

* Difference in HbA2 levels in three groups was statistically not significant

The table shows retention time of small S-window peaks along with their HbA2 percentages and the frequency of additional cases suspected of β-thalassaemia minor among 192 total cases.

Using a computerized data system of laboratory, HPLC chromatograms preceding the identified cases were checked for diagnosis of HbS to exclude a carryover effect.

Data entry and analysis was done using the Statistical Package for Social Sciences (SPSS-14.0). Descriptive statistics were calculated which included maximum and minimum values, and means ± one standard deviation for all continuous data. Means were compared using independent sample t test, among groups with different proportions of S-window peaks. A P- value of <0.05 was considered significant. All data was kept anonymous and the study was approved by institutional ethical review committee.

For characterization of the mutation at molecular level, we prospectively collected blood samples of suspected cases of HbA2'. We amplified a 730 bp fragment of δ globin gene by PCR using a forward primer (5'ATCTCAGGGCAAGTTAAGGG-3') at nucleotide positions -150 to -131 from the cap site and a reverse primer (5'AATGTGGGAGAAGAGCAGGT-3') at positions 68-87 from the second intron of δ globin gene [3]. The mutation was verified by direct nucleotide sequencing of the amplified fragment as well as its digestion with C*fo*I restriction enzyme. C*fo*I recognizes and cleaves a site created by the mutation of GGC → CGC at codon 16 to generate two fragments of 480 and 250 bp.

## Results

We reviewed 10186 chromatograms in the year 2006 identifying various hemoglobin variants as follows: β -thalassaemia trait, β -thalassaemia major, Hb S disorders, Hb Q and Hb D in 319(3%), 159(1.5%), 14(0.13%), 8(0.07%) and 7(0.06%) samples respectively. Another 192 cases (1.89%) with S-window peaks of <4% were observed with retention time ranging from 4.32-4.62 minutes (mean 4.59 ± 0.05 minutes). While most of our cases had retention times between 4.55-4.62 minutes as reported in literature [14], we also observed six cases with retention times of 4.32-4.44 minutes, and 10 cases with retention times of 4.52-4.54 minutes. Table 1 reviews frequency of potential HbA2' cases with respect to various proportions (<1, 1-2 and >2%) of hemoglobin variant. The table shows that 123 cases (64.2%) had S window peaks of less than 1%. Retention time and levels of HbA2 were not statistically different in these three groups of samples with potential HbA2' mutation.

### Hemogram evaluation

Complete blood counts were available for 163 cases only (Table 2). However owing to missing data the correct diagnosis for anemia can be made for 149 patients only. There were 20 subjects (6 males and 14 females) who were not anemic with mean hemoglobin respectively as 14.1 ± 1.2 and 12.3  ± 0.9 g/dl (data not shown), while 129 subjects (72 males and 57 females) showed hypochromic microcytic anemia with mean hemoglobin of 7.7 ± 2.9 and 7.8 ± 2.2 g/dl in that order.

**Table 2:** Haematological parameters in 163 cases with varying small s- window peaks

S- Window Peaks (N)	Hb (g/dl)	Hct (%)	RBC (10^12^/l)	MCV (fl)	MCH (pg)
<1% (105)	9.5 ± 2.5	30.5 ± 7.4	4.6 ± 1.0	66.6 ± 10.8	21.0 ± 4.6
1-2% (53)	6.5 ± 2.4	21.6 ± 6.9	3.5 ± 1.2	63.2 ± 12.0	19.2 ± 5.0
>2%(5)	4.8 ± 3.1	15.1 ± 9.3	2.0 ± 1.1	75.3 ± 14.8	24.1 ± 6.3

**All cases (163)**	**8.4 ± 2.9**	**27.1 ± 8.6**	**4.2 ± 1.3**	**65.8 ± 11.5**	**20.5 ± 4.9**

The table shows haemoglobin, haematocrit, red cell counts and red cell indices in 163 cases out of 192 total cases where these parameters were available.

### HPLC Chromatogram Analysis

For all cases, mean HbA2 level was 3.19 ± 1.30% (range 1.0-7.0%) and mean S-window level was 0.92 ± 0.48% (range 0.3-3.5%). The observed range for total HbA2 (HbA2+S-window) was 1.60-8.70%.

**Figure 1:**
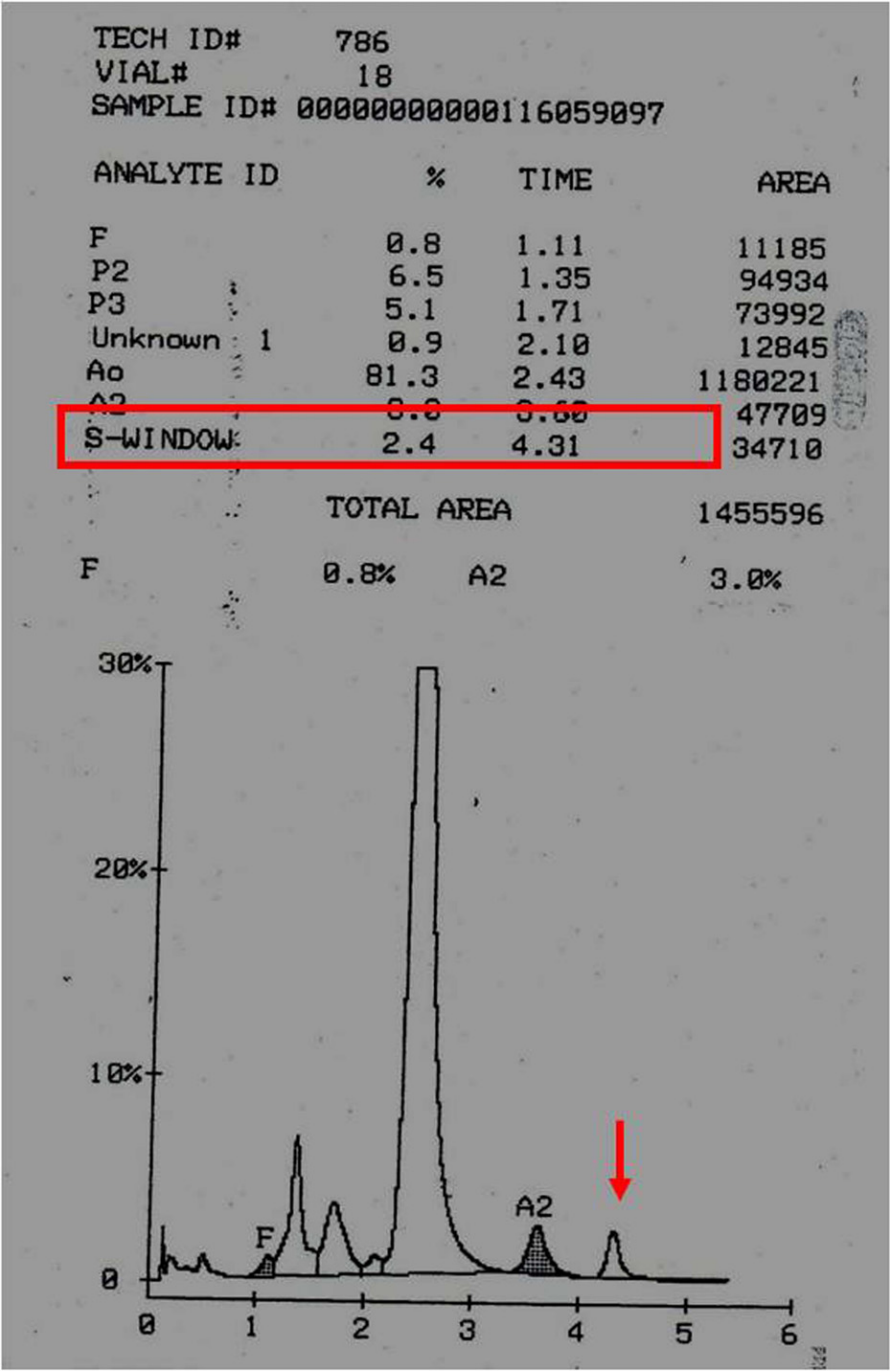
**Chromatogram with suspected HbA2'**. Chromatogram in a patient with S-window peak of 2.4% (red arrow) and Hb A2 of 3.0% (Please note that this chromatogram was not included in this study as it was identified outside the study duration, i.e. after 2006).

During the study period, 319 samples were identified to have β-thalassaemia trait with HbA2 >3.5%. When the results were computed taking account of S-window peaks as presumed HbA2', an additional 64 new cases were suggested of having β -thalassaemia trait. This was before the genetic analysis was performed. Figure 1 shows chromatogram from a patient with S window peak.

### Genetic Analysis

**Figure 2:**
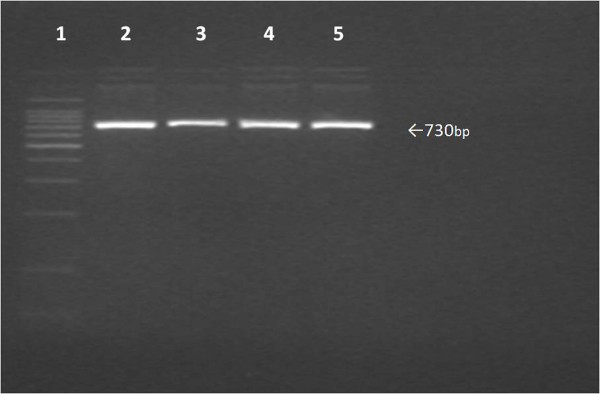
**RFLP-PCR**. Gel electrophoresis of PCR products after C*foI *digestion. Lane1: size marker (100 bp). Lane 2: wild type showing 730 bp fragments. Lanes 3-5: 730 bp of uncleaved amplified fragments from samples with S window peak. Note that C*foI *cleaves the GCG/C site created by HbA2' in delta gene of hemoglobin.

**Figure 3:**
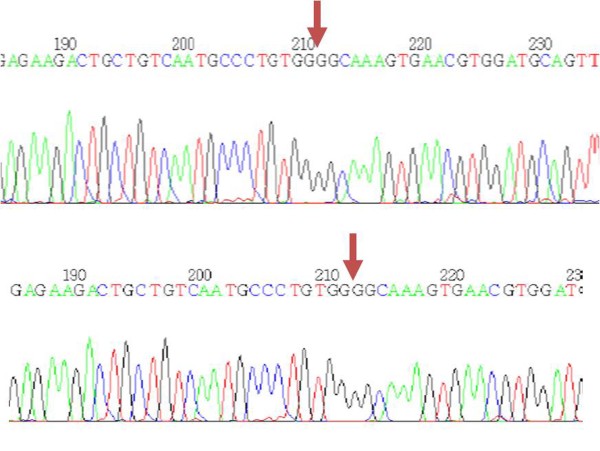
**Gene Sequencing**. DNA sequence derived from a healthy control (upper) and from a patient with S window peak (lower). The sequencing chromatogram shows the same sequence in both individuals [GCCCTGTGGGGCAAAGTGAA] with the arrows indicating the peaks where HbA2' mutation was expected.

To confirm that these S-window peaks represent HbA2', we performed the RFLP based PCR on 13 blood samples, with S-window peaks ranging from 0.6 to 2.4%. A fragment of 730 bp was amplified in each case but the overnight incubation with the enzyme C*fo*I failed to digest the PCR products in any sample (Figure 2). Direct nucleotide sequencing confirmed the absence of HbA2' mutation in these thirteen samples (Figure 3). The entire 730 bp amplified fragment, containing the first two exons of delta globin gene, was reviewed for any other delta gene mutations, but was found to resemble the wild type gene in all the cases.

### Discussion of findings

This study gives an insight into the reliability of HPLC for detecting HbA2' cases in Pakistani population. HPLC has been shown to be a reliable instrument for the detection of HbA2' in many parts of the world [6,13,14]. We found that the trends (retention time etc.) observed on HPLC chromatogram analysis in this study were similar to that reported for HbA2' in previously published studies. However, we establish no evidence of the existence of HbA2' mutation in Pakistani population.

Here we compare the findings of our HPLC chromatogram analysis with previously published studies. A mean retention time of 4.59 ± 0.05 minutes for HbA2' cases was observed in the study which was comparable to other reports [13,14]. An HPLC chromatogram displaying HbA2' with retention time of 4.41 minutes was reported by Van Kirk et al [14]. We also observed a few cases with lower than average retention times. Retention time of a hemoglobin variant can change with different lots of reagents (including the ion-exchange column)[15]. The levels of HbA2 were found to be higher than those of HbA2' in all cases similar to other published reports [2,6,14,16]. All cases with suspected HbA2' of >2% fell under the category of β-thalassaemia minor if S-window peak was taken into account, which was also consistent with previously published studies [10,14]. Despite the similarities of our HPLC chromatogram analysis with HbA2'studies published earlier, the mutation was absent on genetic analysis.

Sixty four percent cases in our study showed S-window peaks of less than 1%. Such low peaks (up to 0.6%) have been reported earlier but were never confirmed at molecular level [6,14]. Retention time of these cases was also very similar to that reported for HbA2' diagnosis by chromatography. However, HbA2' mutation was not observed on molecular analysis in any of our analyzed patients.

In the absence of HbA2', what may have caused these peaks? Many studies have reported double heterozygotes with HbA2' levels of >2% [2,6] and as high as 3.3% [10]. Blood transfusions in sickle cell disease may dilute HbS to as low as 2.6% [14]. Therefore it is advisable to carefully evaluate S-window peaks of 2-4% in relation to clinical details and genetic analysis to exclude sickle cell disease. But authors believe that as HbS elutes at 4.51 min [13] and all our cases except six had retention time greater than 4.52 min, HbS was not expected in our samples. Moreover, a peak of this size in S-window can also be due to carry over, which was however excluded in our study by careful inspection of previous samples' results for having hemoglobin S. Hb Manitoba and Montgomery have HPLC retention time (4.58 min) identical to that of HbA2', but they are easily identified by their higher percentages (15.7 to 16.5%) [13]. Eng et al reported cases with a variant of HbA2 with same mobility on starch gel electrophoresis as HbA2'[17]. This was designated as HbA2-Indonesia and was later found to be *α*_2_*δ*_2_^16 Gly → Arg ^[18]. In our study, nucleotide sequencing of the amplified DNA segment excluded all mutations in the first two exons of delta globin gene. There is a possibility that some of these small peaks may be due to α chain variants having a thalassaemia phenotype, or a novel mutation. As many as 30 hemoglobin variants elute in S-window on HPLC with overlapping retention times[15]. Unfortunately, because of limited funds, we could not go into this depth of analysis.

Methemoglobin produces a peak similar to that of a delta chain variant on HPLC and hinders chromatogram interpretation [19]. Hemoglobin in blood samples may spontaneously oxidize to methemoglobin secondary to inherited or acquired cause. Inherited causes are associated with high hematocrit which was not seen in any of our patients, and acquired methemoglobinemia is a life threatening condition. Thus, indirect evidences exclude this possibility. Laboratory error may also be responsible for such peaks in our study, and an attempt will be made at recalibrating the instrument, before proceeding with further genetic studies. Authors believe that samples with small S-window peaks should be reanalyzed by HPLC to see the consistency in appearance of these peaks. In either case, it is of critical importance to ensure the presence of HbA2' at molecular level.

In Pakistan, the diagnosis of β-thalassaemia, whether major or minor bears a social stigma. Hence, we must exercise an utmost care when diagnosing this condition and should do so at the highest level of certainty. In the years to come, many other laboratories in Pakistan will acquire HPLC. Only a few places have the resources to perform genetic analysis. In our study, 64 additional cases would have been labelled as β-thalassaemia minors as per identification of HbA2' by HPLC, if genetic analysis was not performed. Hence, we report our negative results to avoid future diagnostic dilemmas in underprivileged laboratories and prevent incorrect labeling of patients as β-thalassaemia minors.

### Strengths and Limitations

This is the first report that addresses the suspected HbA2' cases from Pakistan. An attempt was made to determine the true nature of these peaks by PCR amplification and nucleotide sequencing. As AKUH laboratory receives blood samples from all over the country our results are expected to represent the entire population.

One of the weaknesses of the study was failure to identify the true nature of these small S-window peaks including evaluation of exon3 of delta globin gene. The peaks could be equally due to a novel delta chain variant or α-thalassaemia with a similar retention time.

## Conclusions

HbA2' was not detected in potential cases identified by HPLC in the Pakistani population. Hence, small S-window peaks obtained on HPLC should not be reported as HbA2' unless confirmed at the genetic level. Routine genetic studies are not cost effective and thus, not recommended. We recommend further larger molecular studies to determine the true nature of these peaks.

## Competing interests

The authors declare that they have no competing interests.

## Authors' contributions

MN participated in the design of the study and performed the statistical analysis and wrote the manuscript. BM participated in its design and coordination and helped to draft the manuscript. AN carried out the molecular genetic studies, participated in the sequence alignment and drafted part of the manuscript. MRH performed chromatographic analysis and participated in data collection and study designing. All authors read and approved the final manuscript
